# Optimization of Pulsed Laser Energy Density for the Preparation of MoS_2_ Film and Its Device by Pulsed Laser Deposition

**DOI:** 10.3390/mi15080945

**Published:** 2024-07-24

**Authors:** Wei Cai, Yuxiang Liu, Rihui Yao, Weijian Yuan, Honglong Ning, Yucheng Huang, Shaojie Jin, Xuecong Fang, Ruhai Guo, Junbiao Peng

**Affiliations:** 1 Ji Hua Laboratory, Foshan 528000, China; caiwei@jihualab.ac.cn; 2 Guangdong Basic Research Center of Excellence for Energy & Information Polymer Materials, State Key Laboratory of Luminescent Materials and Devices, School of Materials Sciences and Engineering, South China University of Technology, Guangzhou 510640, China; lyx19924688627@163.com (Y.L.); yaorihui@scut.edu.cn (R.Y.); wjyuan163@163.com (W.Y.); yucheng_h@163.com (Y.H.); 18770602788@163.com (S.J.); fxc20020122@163.com (X.F.); psjbpeng@scut.edu.cn (J.P.); 3 GDJH Advanced Display Equipment Ltd., Foshan 528000, China

**Keywords:** MoS_2_, pulsed laser deposition, pulsed laser energy density, heterojunction

## Abstract

This article aims to explore the most optimal pulsed laser energy density when using the pulsed laser deposition (PLD) process to prepare the MoS_2_ films. We gradually increased the pulsed laser energy density from 70 mJ·cm^−2^ to 110 mJ·cm^−2^ and finally determined that 100 mJ·cm^−2^ was the best-pulsed laser energy density for MoS_2_ films by PLD. The surface morphology and crystallization of the MoS_2_ films prepared under this condition are the best. The films consist of a high-crystallized 2H-MoS_2_ phase with strong (002) preferential orientation, and their direct optical band gap (E_g_) is 1.614 eV. At the same time, the Si/MoS_2_ heterojunction prepared under the optimal pulsed laser energy density shows an opening voltage of 0.61 V and a rectification ratio of 457.0.

## 1. Introduction

In recent years, two-dimensional materials such as graphene, transition metal sulfide (TMDCs), black phosphorus, and hexagonal boron nitride (h-BN) have been widely used in the field of optoelectronics [1,2,3,4]. The typical TMDCs material MoS_2_ is one of the most commonly used and promising two-dimensional semiconductor materials. With its adjustable optical band gap and unique photoelectric characteristics, MoS_2_ has shown promising application prospects in the fields of transistors, photoelectronic devices, and energy storage devices [5,6,7].

Given the excellent properties of MoS_2_, researchers have developed several methods for preparing MoS_2_ films of different thicknesses. The mechanical stripping method uses a special tape to overcome the van der Waals force between MoS_2_ layers repeatedly peeling off the entire material, and finally obtaining a single layer to hundreds of layers of MoS_2_ nanosheets [8]. The method is simple to operate and has a high stripping speed. However, the shape and thickness of the peeled nanosheets are uncontrollable, the efficiency of this preparation is low and the repeatability is poor. Therefore, this method is mainly used in the laboratory [9]. Chemical vapor deposition (CVD) is another method that is relatively easy to achieve large-scale production of MoS_2_ films. This method uses transition metal (Mo) or transition metal oxide (MoO_3_) to prepare MoS_2_ by oxidation–reduction reaction with sulfur (S) [10,11,12]. However, CVD still has challenging problems in terms of thickness control, purity, and structure/morphology uniformity [13]. Therefore, it is currently necessary to find a method that can produce large-area MoS_2_ films at relatively low temperatures [14,15,16].

Pulsed laser deposition (PLD) is a technology that uses pulsed laser to bombard the target and deposit the bombarded plasma on the substrate for film growth. PLD is known for its strong process latitude. By accurately and independently controlling the growth process parameters such as the frequency and energy of the pulsed laser and background pressure, it is possible to produce high-quality MoS_2_ films from a few nanometers to a few microns in thickness. By accurately and independently controlling the growth process parameters such as the frequency and energy of the pulsed laser and background pressure, it is possible to produce high-quality MoS_2_ films from a few nanometers to a few microns in thickness. Tumino [17] et al. studied the surface properties of single-layer MoS_2_ deposited on Au (111) by PLD, starting from the growth of MoS_2_ nanocrystals to the formation of a single-layer film uniformly covering the substrate surface on the centimeter scale. Siegel et al. [18] have demonstrated the growth of high-quality monolayer and few-layer MoS_2_ films on transparent sapphire substrates using a PLD technique. The number of MoS_2_ monolayers in the films was very precisely controlled by varying the number of pulsed laser pulses. Serna et al. [19] found a scalable and catalyst-free method to deposit MoS_2_ films over large areas on a wide range of substrates without any additional surface preparation, including single-crystal (sapphire and quartz), polycrystalline (HfO_2_), and amorphous (SiO_2_) substrates. Tumino and Siegel successfully achieved the precise control of MoS_2_ films’ thickness on the gold substrate or transparent sapphire substrates by controlling the process parameters of PLD, but the studies about MoS_2_ films deposited on the silicon substrates by PLD are few relatively.

Apart from the thickness of MoS_2_ film, there is no clear demonstration of the relationship between the properties of the film and the process parameters, but this one is actually crucial. Increasing the energy density of the pulsed laser usually results in more target materials being vaporized and increasing the growth rate of the film. However, high energy density may lead to the spatter of large particles and damage the uniformity and smoothness of the films. The lower frequency of the pulsed laser slows down the growth rate of the films, which is favorable for improving the crystal quality, but the growth time will be prolonged. Higher substrate temperature helps to increase the surface mobility of the deposited atoms and promotes crystal growth, which improves the crystallinity and orientation of the film.

There are many controllable process parameters for films’ quality, and the strategy of preparing MoS_2_ films by PLD still needs further research. Thus, a systematic analysis of the process parameters for PLD depositing MoS_2_ films is required. The effect of varying a process parameter during the deposition process on the properties of films and devices is determined by the control variable method. Since the laser energy density mainly determines the generation of the plume and the deposition process, this work chooses to find the relationship between laser energy density and the properties of films and devices. In this paper, we have been able to identify an optimal pulsed laser energy density (W_PL_) for preparing highly crystallized MoS_2_ films exhibiting a direct bandgap of 1.614 eV and a very high photoelectric performance.

## 2. Results and Discussion

We used PLD to prepare different MoS_2_ films on the silicon substrate by setting W_PL_ = 70 mJ·cm^−2^, 80 mJ·cm^−2^, 90 mJ·cm^−2^, 100 mJ·cm^−2^ and 110 mJ·cm^−2^, respectively. All other process parameters were consistent (the distance between the target and the substrate was 7.5 cm, the deposition pressure was 5 × 10^−4^ Pa, the pulsed laser frequency was 5 Hz, the number of laser pulses was 3000 and the substrate temperature was 400 °C).

**Figure 1:**
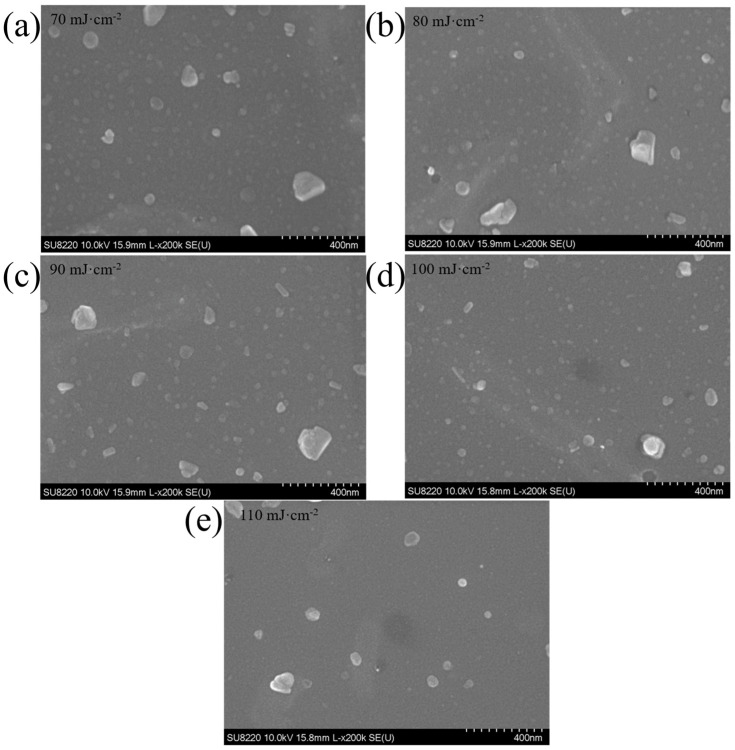
SEM images of MoS_2_ films deposited with different W_PL_ ((**a**) 70 mJ·cm^−2^, (**b**) 80 mJ·cm^−2^, (**c**) 90 mJ·cm^−2^, (**d**) 100 mJ·cm^−2^ and (**e**) 110 mJ·cm^−2^).

Figure 1 shows the surface morphology of MoS_2_ films under a scanning electron microscope (SEM). With W_PL_ increasing, the uniformity of films gradually improved and the irregular bulk particles on the surface gradually disappeared. The thickness and density of the films were measured using X-ray reflectivity (XRR) and the roughness of the film’s surface was measured using an atomic force microscope (AFM), the data are shown in Table 1. The thickness of films increased with laser energy density increasing from 70 mJ·cm^−2^ to 100 mJ·cm^−2^. However, the films’ thickness decreased to 9.938 nm sharply and was smaller than the films with W_PL_ = 70 mJ·cm^−2^ when the W_PL_ was 110 mJ·cm^−2^. The higher W_PL_ on the MoS_2_ target leads to a stronger bombardment effect, the escaped atoms from the target’s surface have higher energy, and the collision effect between these atoms is more intense when atoms reach the substrate. When the energy is too high (110 mJ·cm^−2^), the escaping atoms have so high kinetic energy that knocks away the films already deposited on the substrate, so the thickness of the films decreases dramatically, and the roughness becomes larger.

Thus, the W_PL_ should be chosen under 110 mJ·cm^−2^. In addition, it can be seen from Table 1 that the films prepared at 100 mJ·cm^−2^ have the smallest roughness, indicating that the film’s surface morphology is the best, and thus 100 mJ·cm^−2^ is a good choice.

**Figure 2:**
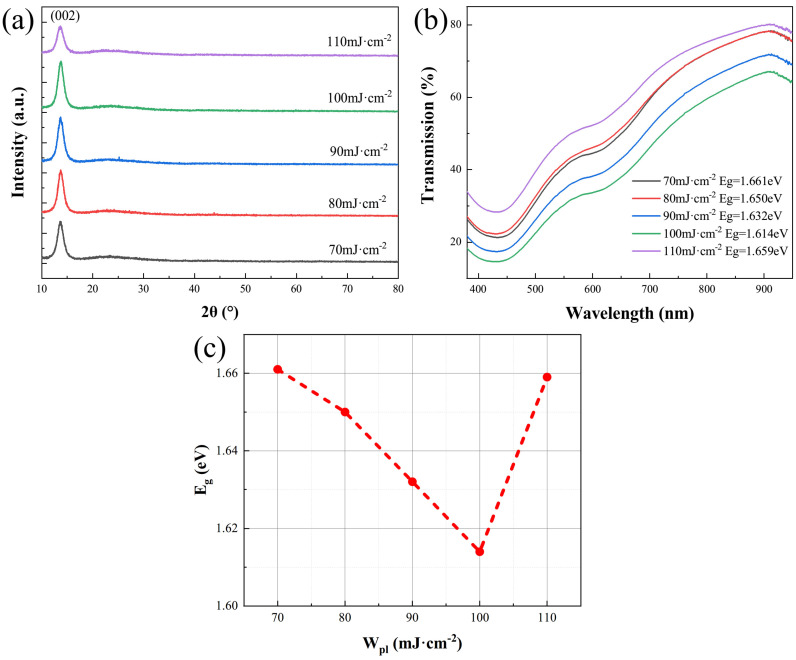
(**a**) XRD patterns of MoS_2_ films deposited with different W_PL_. (**b**) Optical transmission spectrum of MoS_2_ films deposited with different W_PL_ in visible region. (**c**) Calculated E_g_ of MoS_2_ films deposited with different W_PL_.

X-ray diffraction (XRD) test was performed on MoS_2_ films deposited with different W_PL_ to characterize their crystallization and the results are shown in Figure 2a. All kinds of MoS_2_ films have sharp diffraction peaks at 2θ = 13.66°, corresponding to the (002) crystal face of the hexagonal (2H) phase MoS_2_. When W_PL_ increased from 70 mJ·cm^−2^ to 100 mJ·cm^−2^, the intensity of the diffraction peak increased gradually. However, when W_PL_ increased to 110 mJ·cm^−2^, the intensity of the diffraction peak decreased. This shows that when W_PL_ is 100 mJ·cm^−2^, the crystallization of MoS_2_ films is best.

Figure 2b shows the optical transmission spectrum of MoS_2_ films deposited on the quartz substrates with different W_PL_ in the visible region. The spectrum clearly shows that MoS_2_ films absorb more light at shorter wavelengths and have the strongest absorption waves at 430 nm. Combining the transmission spectrum and Tauc equation ((αhv)1m=B(hv−Eg), where *α* is the absorption coefficient, *m* is directly related to the semiconductor type, generally using m = 1/2 in calculating direct band gap), we calculated the direct optical band gap (E_g_) of MoS_2_ films as shown in Figure 2c. As W_PL_ increased from 70 mJ·cm^−2^ to 100 mJ·cm^−2^, E_g_ decreased significantly from 1.661 eV to 1.614 eV. When W_PL_ increased to 110 mJ·cm^−2^ E_g_ again increased to 1.659 eV. This E_g_ variation is definitely dictated by the crystallinity and most importantly the nanostructural arrangement of the MoS_2_ films, which is consistent with the above XRD patterns. The E_g_ has a great influence on the optical properties of materials. In general, materials with smaller E_g_ have a wider absorption range for light waves. Therefore, the MoS_2_ films prepared by W_PL_ = 100 mJ·cm^−2^ are more advantageous in the application of optoelectronic devices.

**Figure 3:**
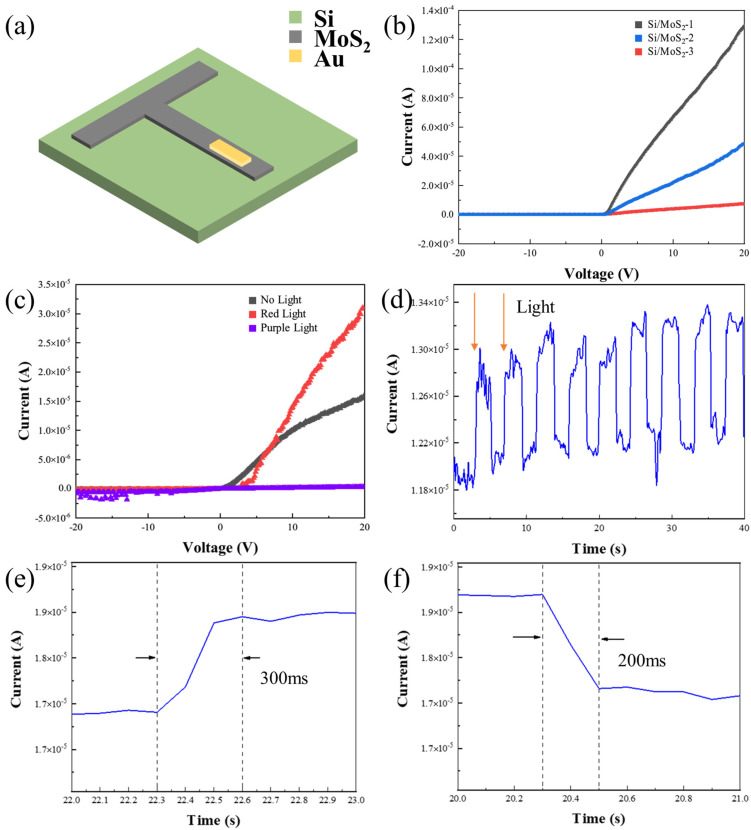
(**a**) The structure of Si/MoS_2_ heterojunctions. (**b**) The I-V characteristic of Si/MoS_2_ heterojunctions with different thicknesses tested in dark conditions. (**c**) The I-V characteristic of Si/MoS_2_ heterojunctions under different lights. (**d**) The cyclic optical response curve of Si/MoS_2_ heterojunctions (**e**) The optical response time test curves of Si/MoS_2_ heterojunctions. (**f**) The optical recovery time test curves of Si/MoS_2_ heterojunctions.

The above test results show that W_PL_ = 100 mJ·cm^−2^ is the most suitable setting for preparing MoS_2_ films by PLD. Therefore, we used PLD to deposit MoS_2_ film and a gold electrode on Si substrates to obtain Si/MoS_2_ heterojunctions. The structure of the heterojunctions is shown in Figure 3a. By setting W_PL_ = 100 mJ·cm^−2^ and changing the number of laser pulses (1000, 3000, 5000), we could obtain MoS_2_ films with different thicknesses. According to the different thicknesses of deposited MoS_2_ films (2.0 nm, 4.1 nm and 4.7 nm), the heterojunctions were named Si/MoS_2_-1, Si/MoS-2, and Si/MoS_2_-3, respectively.

The electrical properties of Si/MoS_2_ heterojunctions were studied under dark conditions and the test results are shown in Figure 3b. It can be observed that the Si/MoS_2_ heterojunctions possess a characteristic of unilateral conductivity. The opening voltage (V_on_), forward current (I_d_^+^), reverse current (I_d_^−^), and rectification ratio (A) data of three kinds of samples are listed in Table 2. The A of Si/MoS_2_-3 is only 67.7 and its electrical performance is poor. The A of Si/MoS_2_-1 and Si/MoS_2_-2 is 457.0 and 467.6, respectively. The V_on_ of Si/MoS_2_-1 is 0.61 V, which is smaller than that of Si/MoS_2_-2, so the electrical performance of Si/MoS_2_-1 is relatively better. The reason why MoS_2_ films show P-type semiconductor properties is the generation of S-vacancy. Therefore, under certain conditions, the more carriers the films can provide with more defects, the better the conductivity of the device. The films of Si/MoS_2_-1 are the thinnest and have more defects, which shows the best electrical properties.

Subsequently, we tested the photoelectric performance of the Si/MoS_2_ heterojunctions, and the results are shown in Figure 3c. It can be clearly observed that the response of Si/MoS_2_ heterojunctions under red light and violet light was different from that under dark conditions. The reason is that the charge separation phenomenon occurred in Si/MoS_2_ heterojunctions and resulted in photogenerated charge carriers under light. Compared with dark conditions, I_d_^+^, I_d_^−^, and V_on_ of heterojunctions increased significantly under red light conditions. This is because the photogenerated carriers produced after illumination increased the diffusion current concentration, breaking the balance between multiphoton diffusion and minority drift, and the drift current concentration began to increase, and the space charge region became wider. At this point, higher voltage was required to fill the increased portion of the space charge region, so V_on_ increased. When V = −20 V, I_d_^−^ = −1.55 × 10^−7^ A. When V = 20 V, I_d_ ^+^ = 3.09 × 10^−5^ A. The V_on_ of Si/MoS_2_ heterojunctions increased to 3.49 V, and the A reduced to 199.35. When exposed to violet light, the Si/MoS_2_ heterojunctions lost their characteristic electrical properties. The reason may be that the energy of violet light is so high that the deep-level impurities became charged centers after ionization, scattering the carriers and reducing the carrier mobility and conductive performance.

Figure 3d shows the cyclic optical response curve of Si/MoS_2_ heterojunctions. When red light was used, the Si/MoS_2_ heterojunctions current increased, and the circulation still remained in the relative range after being repeated several times, which indicated the Si/MoS_2_ heterojunctions possessed good repeatability. Figure 3e,f are the optical response time and recover time test curves of n-Si/MoS_2_ heterojunctions. It can be seen that the response time was 300 ms and the recovery time was 200 ms, so the response speed is relatively rapid.

## 3. Conclusions

In conclusion, we have found the relationship between laser energy density and film properties when preparing MoS_2_ films using PLD technology. When the laser energy density increased within a certain range, the thickness of the MoS_2_ films increased and the optical band gap decreased gradually. When the laser energy density continued to increase until exceeding the optimal laser energy density, the thickness of the MoS_2_ films decreased dramatically and the properties deteriorated severely. Therefore, we confirmed that the optimal laser energy density for the preparation of MoS_2_ films using PLD technology is 100.0 mJ·cm^−2^. The surface shape and crystallization of the MoS_2_ films prepared under this condition are the best. Because the films consist of a high-crystallized 2H-MoS_2_ phase with strong (002) preferential orientation, their direct optical band gap (E_g_) is 1.614 eV and demonstrates excellent photoelectrical properties. At the same time, the Si/MoS_2_ heterojunction prepared under the optimal pulsed laser energy density shows an opening voltage of 0.61 V and a rectification ratio of 457.0.

## 4. Experiment Section

(1) Target and substrates: The MoS_2_ target used in this experiment was purchased from ZNXC Technology Co., Ltd. (Beijing, China). with a target size of 25.4 mm in diameter and 3 mm in thickness, and a purity of 99.99%. The substrates used in this experiment were silicon and quartz substrates, which were purchased from Guangzhou New Vision Optoelectronics Technology Co., Ltd. (Guangzhou, China).

(2) Substrates’ cleaning: Firstly, the silicon and quartz substrates were discharged on a washing rack, and then they were ultrasonicated with isopropyl alcohol for 10 min, deionized water for 10 min each twice, and isopropyl alcohol for 10 min, and finally the cleaned substrates were placed in an oven at 80 °C for drying, and then taken out for use after 1 h.

(3) MoS_2_ film deposition: The laser used in this study was a COMPex201 excimer laser manufactured by Coherent (Saxonburg, PA, USA), with KrF gas as the working gas and an intracavity working air pressure of 3400 mbar. The main wavelength of the output laser was 248 nm, the adjustable range of the pulsed laser frequency was from 1 to 10 Hz and the adjustable range of the laser pulse energy was from 180 to 750 mJ. MoS_2_ target and substrates were placed in the PLD cavity of the No. 1 target position and the sample stage, respectively, the distance between the target and the substrate was 7.5 cm, the deposition pressure was 5 × 10^−4^ Pa, the laser frequency was 5 Hz, the number of laser pulses was 3000 times, the substrates’ temperature was 400 °C. During the deposition process, the MoS_2_ target was rotated while the laser beam was laterally swept across its entire surface to ensure a uniform erosion pattern of the target. This enables a more spatially extended source of ablated species covering more uniformly the substrate holder. Moreover, the substrate holder itself was concomitantly rotated to improve further the thickness uniformity of the films. Prior to each deposition, the MoS_2_ target surface was in situ cleaned by ablating its surface for 5 min. The laser energy density was variable and was set to 70 mJ·cm^−2^, 80 mJ·cm^−2^, 90 mJ·cm^−2^, 100 mJ·cm^−2^ and 110 mJ·cm^−2^, respectively.

(4) Test and characterization: In this study, an X-ray diffractometer is mainly used to obtain the films’ information such as thickness and crystallization. The X-ray diffractometer is Empyrean DY1577 (XRD: Malvern Panalytical Company, Almelo, The Netherlands), with a test voltage of 40 kV, a test current of 40 mA, a test angle of 2θ of 10~80°, a scanning step size of 0.02°, and a dwell time of 1.5 s per step. The transmission spectrum of the films was tested by UV–Vis spectrophotometer, and then the optical bandgap of the films was calculated by extrapolation. The equipment used in this study was a model UV-2600 spectrophotometer from Shimadzu Instruments Ltd. (Columbia, MD, USA). A semiconductor analyzer is used to test and characterize the electrical properties of the heterojunction. The equipment is Agilent 4155C (Agilent Company, Santa Clara, CA, USA), and by measuring the I-V curve of the heterojunction, turn-on voltage, rectification ratio, and reverse current can be obtained to measure the performance of the device.

## Figures and Tables

**Table 1:** Thickness, density, and roughness of MoS_2_ films deposited with different laser energy densities.

Energy (mJ·cm^−2^)	Thickness (nm)	Density (g·cm^−3^)	Roughness (nm)
70	12.984	4.971	3.74
80	12.662	5.173	1.8
90	13.821	5.126	2.9
100	14.026	5.143	1.44
110	9.938	5.210	1.89

**Table 2:** The opening voltage (V_on_), forward current (I_d_^+^), reverse current (I_d_^−^), and rectification ratio (A) of Si/MoS_2_ heterojunctions with different thicknesses tested in dark conditions.

Sample	V_on_ (V)	I_d_^+^ (A)	I_d_^−^ (A)	A
Si/MoS_2_-1	0.61	4.89 × 10^−5^	−1.07 × 10^−7^	457.0
Si/MoS_2_-2	0.66	1.30 × 10^−4^	−2.78 × 10^−8^	467.6
Si/MoS_2_-3	0.54	7.38 × 10^−6^	−1.09 × 10^−7^	67.7

## Data Availability

All the relevant data are included in this published article.
